# Genomic epidemiology of Streptococcus pneumoniae GPSC6: post-vaccine expansion of β-lactam-susceptible serotype 24F in Europe

**DOI:** 10.1099/mgen.0.001697

**Published:** 2026-05-15

**Authors:** Magnus N. Osnes, Arne M. Taxt, Rebecca A. Gladstone, Martha L. Bjørnstad, Daniel Straume, Vegard Eldholm

**Affiliations:** 1 Norwegian Institute of Public Health, Lovisenberggata 6, P.O. Box 222, NO-0213, Oslo, Norway; 2 Norwegian Veterinary Institute, P.O. Box 64, NO-1431, Ås, Norway; 3 Dept. of Biostatistics, Faculty of Medicine, University of Oslo, P.O. Box 1122, NO-0317, Oslo, Norway; 4 Faculty of Chemistry, Biotechnology and Food Science, Norwegian University of Life Sciences, P.O. Box 5003, NO-1432, Ås, Norway

**Keywords:** phylodynamic analysis, invasive pneumococcal disease, pneumococcal population structure, antimicrobial susceptibility profiling, effective population size, whole-genome sequencing

## Abstract

*Streptococcus pneumoniae* serotype 24F has emerged as a major cause of invasive pneumococcal disease in Europe following the introduction of PCV13 yet remains absent from current paediatric vaccine formulations. We investigated the genomic epidemiology and phylodynamics of serotype 24F within Norway by focusing on the dominant strain Global Pneumococcal Sequence Cluster 6 (GPSC6) and placed these isolates in a global context using 972 genomes from 29 countries. GPSC6 is composed of distinct lineages, which we estimate to have diverged around the mid-twentieth century. Before the pneumococcal conjugate vaccine (PCV) era, Lineage 1 was composed predominantly of vaccine serotypes. The past success of Lineage 1 likely came from its antimicrobial resistance (AMR) gained in the 1980s. Since the introduction of PCVs in childhood immunization programmes, Lineage 2 has expanded rapidly, driven by clades with serotypes not covered by the PCVs. A time-calibrated phylogeny indicates that GPSC6-24F originated in the 1980s and expanded rapidly after the introduction of PCV7 in 2006. In Norway, two importations in the period 2007–2009 seeded most local transmissions. The effective population size of GPSC6 declined after PCV7 introduction, followed by expansion of non-vaccine serotypes, notably 24F and 11A. *In silico* AMR profiling revealed that GPSC6-24F isolates likely remain susceptible to *β*-lactams and other antimicrobial classes but are resistant to co-trimoxazole, contrasting the expansion of multidrug-resistant GPSC10-24F reported from high-antibiotic-use settings. Vaccine-driven selection rather than AMR seems to be shaping the GPSC6-24F dynamics in Europe and highlights the need for continued genomic surveillance, as 24F is not covered by paediatric PCV formulations.

**Impact Statement**

*Streptococcus pneumoniae* is a leading cause of illness and death in children worldwide. While antimicrobial resistance has reduced treatment options for some pneumococcal strains, pneumococcal conjugate vaccines (PCVs) have greatly lowered the burden of invasive pneumococcal disease by targeting the most common disease-causing serotypes. However, after PCV introduction, non-vaccine serotypes and the lineages that carry them have increased in frequency. Continued reduction of pneumococcal disease therefore depends on tracking not only serotypes but also the genetic lineages behind them and the traits that affect their success, including antimicrobial resistance. In this study, we use genomic epidemiology to describe the phenotypic diversity, evolution and spread of the globally distributed strain Global Pneumococcal Sequence Cluster 6, and we place the expanding serotype 24F clade within this broader genetic background. Our paper shows that vaccine-driven shifts in the pneumococcal population can favour non-vaccine serotype clades within established strains and highlight the value of integrating whole-genome sequencing into routine surveillance to inform future vaccine updates.

## Data Summary

### Newly generated sequence data

Raw reads for the newly sequenced Norwegian GPSC6-24F isolates have been deposited in the European Nucleotide Archive (ENA) under study accession PRJEB105365 (secondary ERP186568). Per-sample accessions are provided in the Supplementary Data.

### Reference genome

The hybrid long-/short-read GPSC6 reference assembly generated for this study is available at ENA accession PRJEB105359.

### Publicly available genomes

Publicly available GPSC6 genomes (*n*=872 total from 29 countries; downloaded 1 June 2023) were obtained from the Global Pneumococcal Sequencing Project; corresponding accessions are listed in the Supplementary Data.

### Supplementary data

Isolate-level metadata for all isolates analysed are archived at Zenodo: https://doi.org/10.5281/zenodo.19039141

## Introduction

*Streptococcus pneumoniae* is a Gram-positive bacterium encapsulated by a polysaccharide capsule, which serves as its primary virulence factor. The bacterium is responsible for a range of diseases, from less severe conditions like sinusitis and otitis media to severe infections such as pneumonia, septicaemia and meningitis. Globally, *S. pneumoniae* remains a major cause of childhood mortality with an estimated 225,000 deaths in children <5 years in 2019 [1], yet pneumococcal conjugate vaccines (PCVs) have substantially reduced severe pneumococcal outcomes, with pneumococcal meningitis declining by ~48–74% in children <5 within 6 years of PCV10/PCV13 introduction [2].

In Norway, the annual incidence of invasive pneumococcal disease (IPD) increased through the late 1990s into the early 2000s, reaching ~1,000–1,100 reported cases annually at its highest levels in 2003–2006 [3]. The 7-valent conjugate vaccine (PCV7) was licensed in the EU in 2001 and introduced into the Norwegian Childhood Immunisation Programme in July 2006. Higher-valent vaccines, PCV10 and PCV13, became available in 2009, and PCV13 replaced PCV7 in the Childhood Immunisation Programme in 2011. In 2022, PCV15 and PCV20 were approved, with the first approved for use in children in March 2024 [4]. The introduction of the 7-valent pneumococcal conjugate vaccine (PCV7) in 2006 and its subsequent replacement with the 13-valent vaccine (PCV13) in 2011 led to a significant decline in IPD cases caused by vaccine-type serotypes across all age groups, through both direct and indirect protection [5]. Following the introduction of PCV7, pneumococcal carriage rates in children remained stable, attributed to serotype replacement with non-vaccine types (NVTs) [6,7]. After the introduction of PCV13, carriage prevalence in children decreased, and fewer NVTs emerged [7], likely reducing overall transmission and indirectly protecting the rest of the population. Following PCV13 implementation, the incidence of IPD caused by NVTs has increased across Europe, reducing the effectiveness of vaccination [8].

*S. pneumoniae* serotype 24F is one of several NVTs that have increased in prominence in the post-PCV13 era. Serotype 24F has been linked to meningitis [9,11] and carries a high disease burden in children and infants. In European Union/European Economic Area (EU/EEA) surveillance data for 2022, serotype 24F accounted for 6.8% of serotyped IPD cases in infants <1 year and 6.6% in children aged 1–4 years [12]. These EU/EEA-wide surveillance data align with the PSERENADE study of ‘mature’ PCV settings where serotype 24F was among the most important non-PCV13 serotypes among children <5 years of age, accounting for 4.6 and 4.1% of IPD cases in countries having implemented PCV10 and PCV13, respectively [13]. In Denmark, serotype 24F IPD increased after the introduction of PCV13, particularly driven by GPSC6-ST162 [14]. Also in Norway, serotype 24F is emerging after the introduction of PCV13 [15,17] and has been associated with an observed increase in infections with trimethoprim and sulphamethoxazole resistance [17]. As of March 2026, Norway still uses PCV13 in their Childhood Immunisation Programme [18] but is expected to transition to PCV15 or PCV20, neither of which includes serotype 24F. Since serotype 24F is not a part of current or anticipated paediatric vaccine formulations, monitoring its epidemiology is crucial. The assignment of strains to Global Pneumococcal Sequencing Clusters (GPSCs) has been instrumental for the characterization of strain composition following the introduction of PCVs [19,20]. Notably, the GPSCs contributing to the proliferation of serotype 24F are not uniform across countries and appear to be influenced by local antibiotic usage patterns and PCV history. In countries with high antibiotic consumption, such as France, Lebanon, Spain and Argentina, the multidrug-resistant (MDR) GPSC10 strain has been the primary driver of serotype 24F expansion [10]. This is in contrast to Denmark and Norway, where lower antibiotic consumption correlates with the prevalence of the penicillin (PEN)- and erythromycin-susceptible GPSC6 strain [10,15]. In Japan, where consumption of erythromycin is high and PEN is low, a different strain, GPSC106, which is resistant only to erythromycin, is the main contributor to the rise in serotype 24 F cases [10,21].

This study aimed to characterize the genomic epidemiology of *S. pneumoniae* GPSC6 as a major contributor to serotype 24F disease, by combining targeted sequencing of Norwegian isolates with available data from the Global Pneumococcal Sequencing (GPS) project. Here, relying on time-resolved phylogenetic analyses, we reconstruct the evolution and cross-border transmission of GPSC6, specifically by identifying GPSC6-24F import events to Norway, and assessing how local and global selective pressures – such as vaccine implementation and antibiotic usage – have shaped the dynamics of this strain.

## Methods

### Norwegian GPSC6 isolates enriched with serotype 24F genomes

An IPD isolate is defined as one obtained from a normally sterile site in a patient. Since 2018, all IPD cases have undergone whole-genome sequencing at the Norwegian Institute of Public Health. We retrieved all IPD isolates from routine sequencing identified as GPSC6 from 2018 until January 2023, totalling 71 isolates. Additionally, we included GPSC6 genomes from the historical dataset presented in Eldholm *et al.* [15], covering the period 1982 to 2018, and totalling 166 isolates. In total, 237 Norwegian GPSC6 isolates were included in this study. Most isolates were obtained from blood (*n*=206), with smaller numbers from cerebrospinal fluid (*n*=12), sputum (*n*=5), pleural fluid (*n*=1) and one nasopharyngeal swab (*n*=1); we classified specimen source as unknown for 12 isolates because the available metadata were unclear. The specimen source data are included in the Supplementary Data. Quellung reaction data revealed that 120 of the isolates belonged to serotype 24F, and among genomes without quellung data, *in silico* serotyping (*see* Genome characterization) identified six isolates belonging to ‘serogroup 24’, which means that SeroBa did not resolve the serotype between 24B, 24C or 24F. Based on unpublished data from the Norwegian Institute of Public Health, GPSC6 accounted for ≈86% of serotype 24F isolates from 2013 to 2022. This observation motivated our focus on GPSC6, as it captures the dominant 24F strain in Norway. Out of the 237 Norwegian GPSC6 isolates, 135 are available in the GPS Database [22], 78 new GPSC6-24F isolates from Norway have been deposited in the European Nucleotide Archive (ENA) under study accession PRJEB105365 (secondary ERP186568), and 22 GPSC6-24F isolates have previously been uploaded to ENA and are listed with accessions in our Supplementary Data.

### International contextual isolates

We downloaded all publicly available genomes identified as GPSC6 from the GPS Database as of 1 June 2023. This collection comprised 735 isolates from 28 different countries, spanning the period from 1997 to 2018. Since GPSC6 was reported as the dominant strain in a study of serotype 24F in Denmark [14], we also included all GPSC6 genomes from that study, accounting for an additional 15 isolates from the period 2007 to 2017. In total, the collection consisted of 972 isolates from 1997 to 2023 spanning 29 countries and 6 continents. Metadata for all isolates are available at https://doi.org/10.5281/zenodo.19039141 [23].

### Genome data preprocessing

Raw Illumina reads were assembled *de novo* using SPAdes v3.13.0 [24], employing the ‘careful’ mode to reduce the number of mismatches and short indels. We assessed the quality of the assembled genomes using QUAST v5.2.0 [25]. Assemblies were filtered to remove contigs with a k-mer coverage of less than 3 and a length of less than 500 nucleotides. One isolate was excluded from downstream analyses due to inconsistencies in genome length and read quality (not included in the total list of isolates in the metadata).

### Genomic characterization

We used Pathogenwatch v23.1.6 [26] to determine GPSCs, identify multilocus sequence types (MLSTs), determine capsule serotypes (with the exception of Norwegian genomes already typed with Quellung reaction) and detect antimicrobial resistance (AMR). Pathogenwatch determines GPSCs using the Global Pneumococcal Sequencing Database (v8) to define strains based on core and accessory genome distance using PoPPUNK (v1.1.0) [27], MLSTs using PubMLST v5.2.0 [28] and serotypes through SeroBa v1.0.1 [29]. For AMR detection, Pathogenwatch utilizes complementary approaches for *S. pneumoniae*. First, Pathogenwatch AMR (v3.0.1) queries assemblies against a curated sequence library [30] using blastn [31] to identify AMR-associated genes and sequence variants linked to resistance across multiple antibiotic classes. Second, the SPN-PBP-AMR pipeline (v0.0.1) predicts *β*-lactam MICs from penicillin-binding protein (PBP) profiles by analysing *pbp1A*, *pbp2B* and *pbp2X* alleles [32,33] and interprets predictions according to the CLSI M100 standard [34]. CLSI defines meningitis (M) and non-meningitis (NM) breakpoints (μg/ml) for the *β*-lactams PEN, amoxicillin (AMO), ceftriaxone (CRO), cefotaxime (CTX), cefuroxime (CXM) and meropenem (MEM) as follows: PEN-M S≤0.06, R≥0.12; PEN-NM S≤2, I=4, R≥8; AMO-NM S≤2, I=4, R≥8; CRO-M S≤0.5, I=1, R≥2; CRO-NM S≤1, I=2, R≥4; CTX-M S≤0.5, I=1, R≥2; CTX-NM S≤1, I=2, R≥4; CXM-NM S≤0.5, I=1, R≥2; MEM-NM S≤0.25, I=0.5, R≥1. Using the *in silico* AMR estimates, we assessed whether isolates were predicted to be MDR, defined as non-susceptibility to three or more distinct antimicrobial classes (described in Supplementary Methods).

### Generation of an appropriate reference sequence for the international collection

To build a phylogeny of the international collection of isolates, a high-quality reference sequence was essential to accurately identify SNPs. We employed PopPUNK v1.1.0 [27] to identify central isolates for the dominant GPSC and to construct a core genome phylogeny. Through manual inspection of mandrake plots [35] at varying perplexity parameter values, as well as the core genome phylogeny, we identified isolate ‘572700’ as an appropriate reference due to its central position among the other isolates. To generate a high-quality reference sequence for this isolate, we utilized a hybrid assembly approach. Long-read data were produced on the Oxford Nanopore GridION platform, and short-read data were generated on the Illumina MiSeq platform. We used Unicycler v0.4.7 [36] to *de novo* assemble a high-quality reference genome by jointly utilizing the long Oxford Nanopore reads and the short, accurate Illumina reads. The resulting assembly was made up of 3 contigs, including 1 major contig of 2,128,511 bp and 2 smaller contigs (24,092 and 566 bp), and is available under ENA study accession PRJEB105359.

#### Reference-based alignment

Genomes were aligned to the reference genome using Snippy v4.6.0 [37]. Snippy utilizes the Burrows-Wheeler Aligner [38] and SAMtools [39] for read alignment across various isolates and leverages FreeBayes [40] for variant identification.

### Filtering recombination events

Recombination events were identified from the core genome alignment consisting of 2,189,054 sites using Gubbins v3.3.0 [41]. IQ-TREE was employed as the tree builder [42], utilizing the GTR+Γ substitution model [43,44]. The clonal frame [45] that remained after the detected recombination regions were removed consisted of 15,857 polymorphic sites. We quantified the relative contribution of recombination to sequence diversification (*r/m*) as the ratio of the number of substitutions attributed to recombination to the number of substitutions attributed to mutation (i.e. substitutions outside inferred recombination segments). We also quantified the relative frequency of recombination versus mutation events using *ρ/θ*. Tree-wide values were calculated as the mean of the per-branch estimates across the phylogeny.

### Dating the GPSC6 tree

Temporal estimates for the GPSC6 phylogeny were obtained using the BactDating v.1.1.1 [46] R package, which utilized information on the recombination events detected with Gubbins. The temporal signal was assessed through a root-to-tip analysis, yielding a $R^{2}=0.37$ (Fig. S1, available in the online Supplementary Material). The one isolate from Kuwait did not have a collection date in the GPS Database. In BactDating, this was handled by specifying the date as missing (NA), in which case it is treated as an unknown date constrained to fall within the range of the other observed collection dates and estimated during the analysis. Multiple molecular clock models were evaluated, including an uncorrelated relaxed clock (RC) [47], an additive RC (ARC) [48] and a continuous ARC [48]. Three independent Markov chain Monte Carlo runs of 10^7^ iterations were performed. We removed the initial 50% of iterations as burn-in and thinned the remaining draws to obtain 10,000 posterior samples. Convergence was evaluated by requiring effective sample sizes >200 and Gelman–Rubin $\hat{R}§amp;lt;1.01$ for all monitored parameters [49]. Node ages were summarized using the posterior median, with 95% credible intervals defined by the 2.5th and 97.5th percentiles. Model comparison was based on the deviance information criterion (DIC) [50]. Using the Akaike information criterion (AIC) thresholds [51] that reportedly work well for DIC [50], the ARC model provided the best fit to the data, as evidenced by ΔDIC>10 over the second-best model. Estimates of node times in the dated tree and credibility intervals are reported for the ARC model.

### Estimating effective population size

The effective population size of the GPSC6 was estimated using two distinct methods: maximum *a posteriori* estimates from Skygrowth v0.3.1 [52] and Bayesian Non-parametric Phylodynamics Reconstruction (BNPR) implemented in the phylodyn v0.9.02 R package [53]. Skygrowth was run with default settings using 50 time intervals (res = 50) and an exponential prior on the precision (smoothing) parameter (tau_logprior = "exponential"). BNPR was run using the default settings, including 100 grid points (lengthout = 100), and gamma priors on the precision parameter (prec_alpha = 0.01, prec_beta = 0.01). Two NVT clades were identified in the global tree: GPSC6-24F and GPSC6-11A, and we estimated the effective population size separately for these clades.

### Geographical transmission dynamics of GPSC6-24F and import analysis to Norway

We reconstructed the geographical states on the nodes of the dated GPSC6-24F clade using maximum likelihood ancestral state reconstruction (ASR) with the symmetrical transition rate model implemented in the ace function in the R package ape v5.0 [54]. Four countries were represented in this clade: Norway, France, Denmark and India. India was represented by a single isolate, and India-specific geographical transition rates were therefore not identifiable. We evaluated a set of alternative parameterizations in which India was assigned rate parameters equivalent to those among Denmark, France and Norway. For each parameterization, the transition rate between India and the location with which it shared rate structure was allowed to take the value of each of the other estimated rates in turn. We repeated the ASR for each alternative parameterization and selected the final parameterization as the one with the lowest AIC. The transition-rate matrix (***Q***) of the model with the lowest AIC was as follows:


$$Q=\left[\begin{array}{ccccc} & \text{Denmark} & \text{France} & \text{India} & \text{Norway}\\ \text{Denmark} & 0 & q_{1} & q_{3} & q_{2}\\ \text{France} & q_{1} & 0 & q_{2} & q_{3}\\ \text{India} & q_{3} & q_{2} & 0 & q_{2}\\ \text{Norway} & q_{2} & q_{3} & q_{2} & 0 \end{array}\right],$$

where $q_{1}$, $q_{2}$ and $q_{3}$ denote distinct estimated transition-rate parameters. Connected geographical groups of nodes estimated to have more than 50% probability of being in the same geographical location were interpreted as continuous transmission in the same geographical location (local transmission), while geographical state changes from one node to a descendant node were interpreted as an importation event. We used LineageHomology [55,56] to count import and local transmissions automatically. We accounted for the estimated uncertainty in the ASR estimates of the nodes by redrawing ancestral states 100 times according to the estimated ancestral state probabilities, and recounting importation and local transmissions for each draw.

### Data analysis and visualization

Apart from the bioinformatics analysis, all analyses were performed in R v4.2.1 [57]. For data processing, we used dplyr v1.1.0 [58] and tidyr v1.3.2 [59]. For data visualization, we used R packages, ape v5.6.2 [54], phytools v1.2.0 [60], ggplot2 v3.4.2 [61] and ggtree v3.4.2 [62].

## Results

### Sample description

**Fig. 1.**
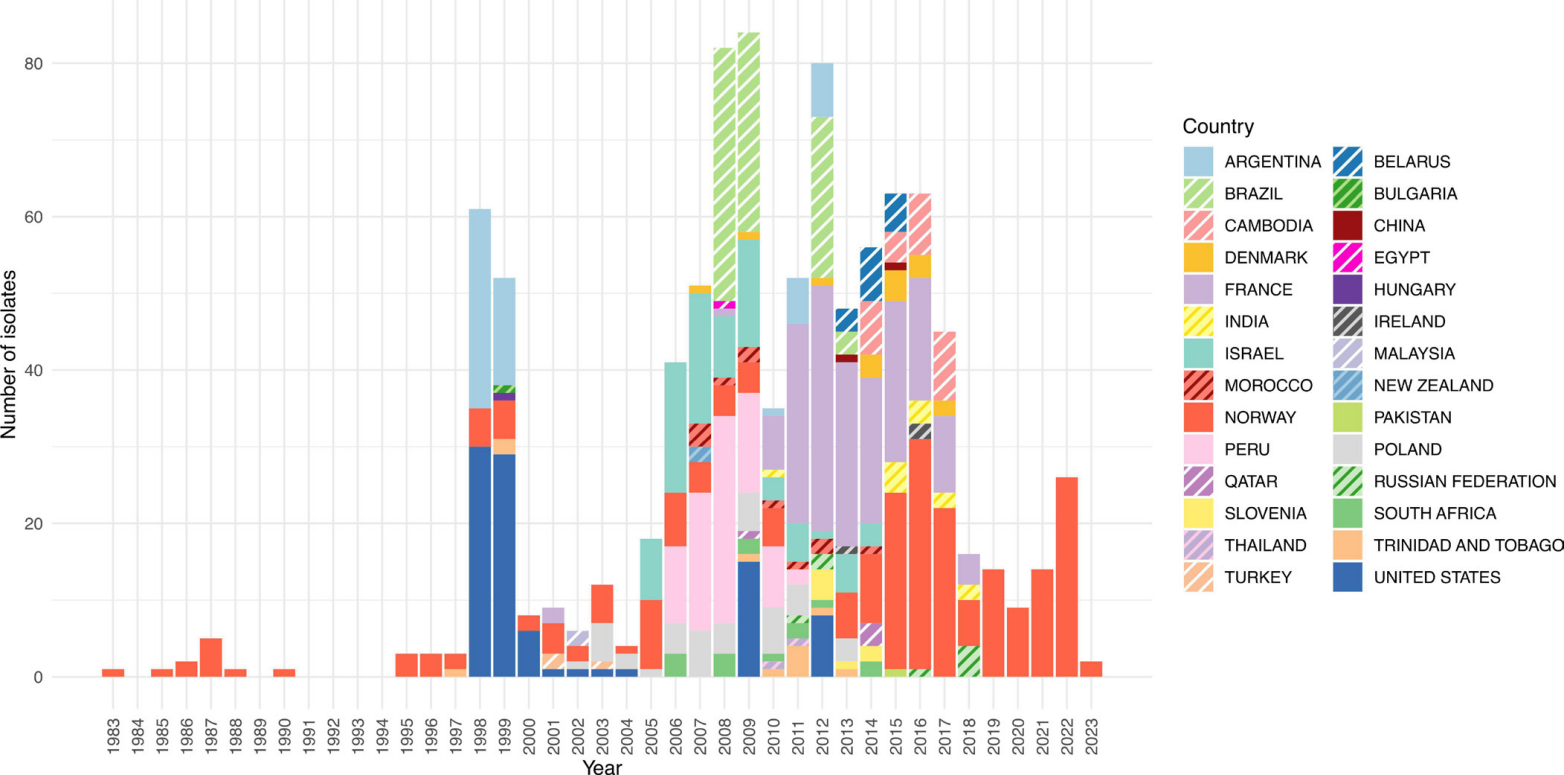
Distribution of collection dates of samples from different countries. One isolate from Kuwait is omitted from this figure because the collection date was not available.

The collection contains data from 29 countries (Fig. 1). The most common locations were Norway (237 out of 972; 24.4%) and France (162 out of 972; 16.7%), followed by the USA (92 out of 972; 9.5%), Brazil (83 out of 972; 8.5%), Israel (81 out of 972; 8.3%) and Peru (78 out of 972; 8.0%). Additional locations include Argentina (54 out of 972; 5.6%), multiple European countries (Poland, 41 out of 972; 4.2%; Denmark and Belarus, 15 out of 972 each; 1.5%), Asia and the Middle East (e.g. Cambodia, 28 out of 972; 2.9%; India, 12 out of 972; 1.2%) and Africa (South Africa, 14 out of 972; 1.4%; Morocco, 11 out of 972; 1.1%; Egypt, 1 out of 972; 0.1%). The oldest genomes are all from Norway because the temporal range of GPS was recently extended by a Norwegian collection that contributes the earliest GPSC6 genomes, including isolates from the 1980s and 1990s [15]; however, the dataset is more geographically diverse from 2008 to 2017.

### Ancestral relationships in GPSC6

**Fig. 2.**
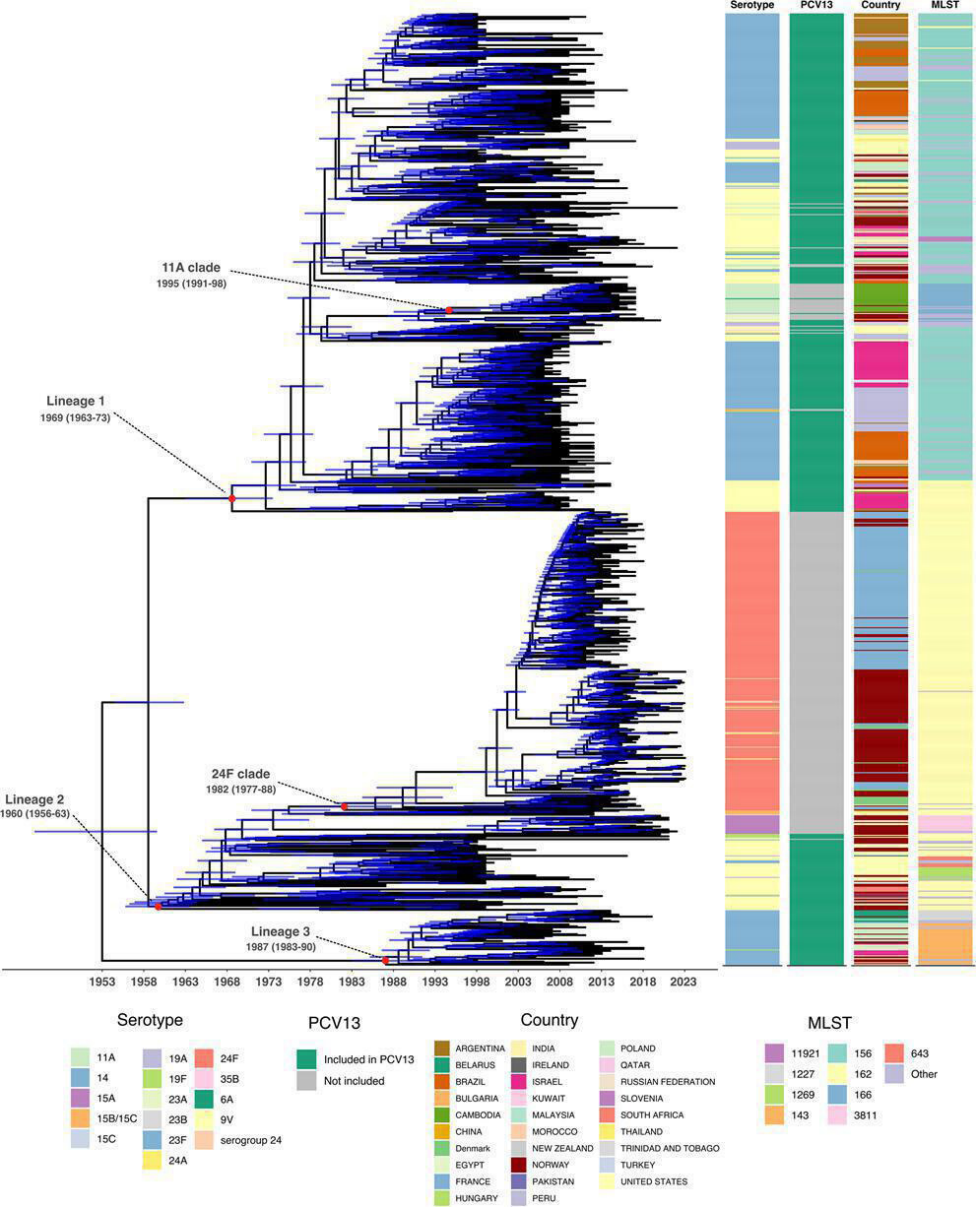
Dated GPSC6 phylogeny with metadata: serotype, PCV13 vaccine coverage, country and MLST. The 95% credibility intervals for the node dates are shown as blue transparent lines. Clades of interest are annotated with Time to Most Recent Common Ancestor (TMRCA) and 95% credibility intervals.

We generated a temporally resolved phylogenetic tree of all the GPSC6 isolates using Gubbins, IQ-TREE and BactDating (Fig. 2). The tree-wide ratio of substitutions attributed to recombination to those attributed to mutation (r/m) was 10.27, and the relative frequency of recombination events to mutation events (*ρ*/*θ*) was 0.171. The phylogeny consists of 16 different serotypes. The tree shows that GPSC6 likely emerged in the mid-twentieth century (root date: 1952.91, CI: 1945.97; 1958.68). The estimated molecular clock rate was 9.23×10^−7^ (95% CI: 8.68×10^−7^, 9.73×10^−7^) substitutions per site per year, corresponding to 2.02 (CI: 1.90–2.13) mutations per genome per year. Our estimated clock rate is approximately half the estimate of 1.71×10^−6^ per site per year reported by D’Aeth *et al*. [63] for PMEN3/GPSC6. The difference between the estimated clock rates may reflect differences in the datasets included, as our dataset includes clades not represented in D’Aeth *et al.* [63], such as the 24F clade which comprises 31% of our dataset.

**Fig. 3.**
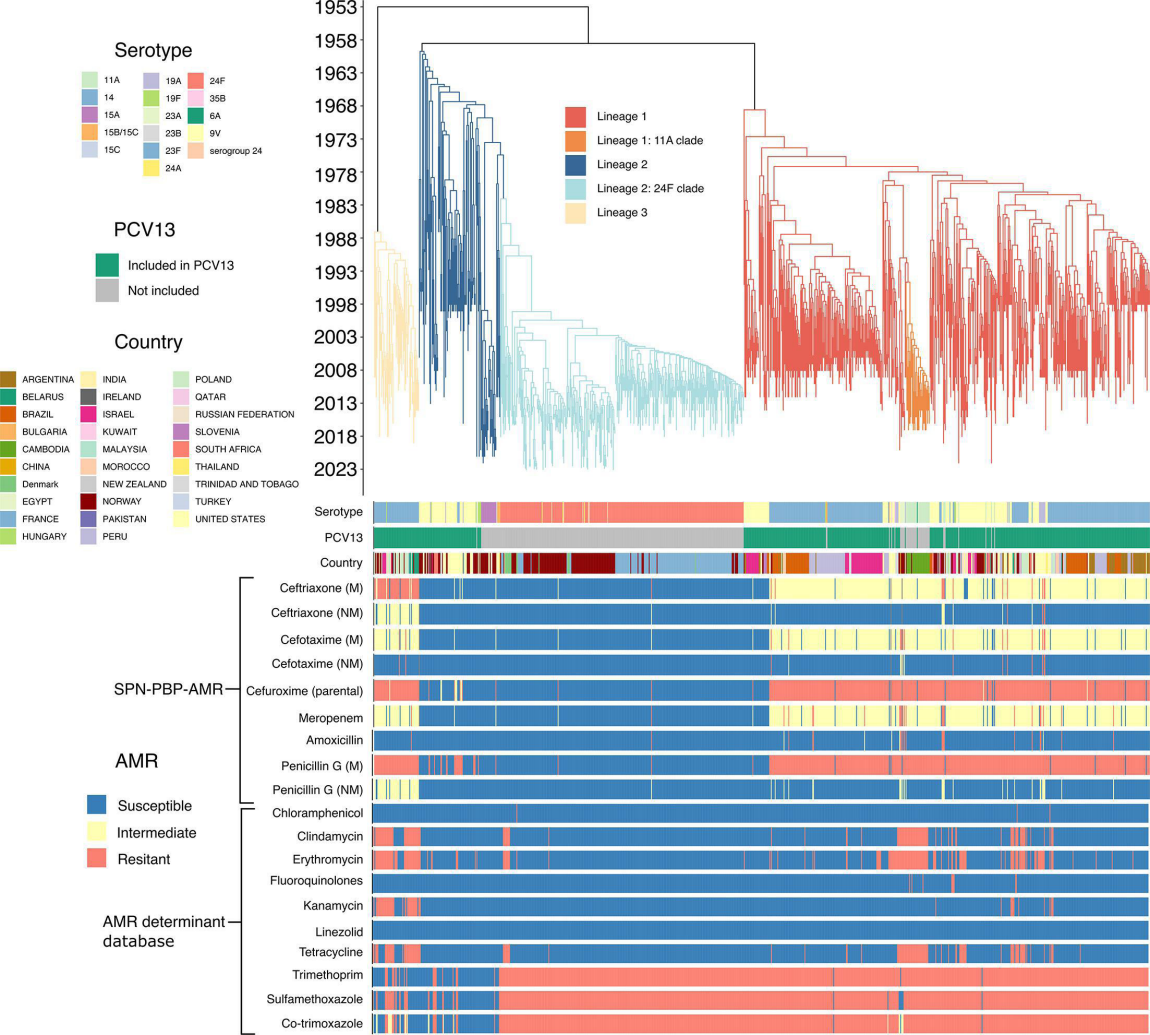
Time-scaled phylogeny of GPSC6 with *in silico* predicted AMR. The annotation fields below the phylogeny display the serotype, PCV13 inclusion status, geographical location and AMR. *β*-Lactam phenotypes (CRO, CTX, CXM, MEM, AMO and PEN G) were predicted using Pathogenwatch’s SPN-PBP-AMR module from PBP profiles (*pbp1A*, *pbp2B* and *pbp2X*) and interpreted using CLSI M100-ed35 breakpoints. Separate meningitis (M) and non-meningitis (NM) predictions are shown where defined in CLSI M100-ed35; otherwise, a single line is shown. Non-*β*-lactam phenotypes were predicted using the Pathogenwatch AMR pipeline based on the detection of known resistance determinants. AMR phenotypes are shown as susceptible (S), intermediate (I) or resistant (R).

GPSC6 is composed of distinct lineages here termed Lineage 1, Lineage 2 and Lineage 3 (Fig. 2) with estimated TMRCAs as follows: Lineage 1: 1968.5 (95% CI: 1962.9, 1973.5), Lineage 2: 1959.7 (95% CI: 1955.7, 1963.5) and Lineage 3: 1987.0 (95% CI: 1983.0, 1990.3). Lineage 1 is diverse, encompassing 10 serotypes and 52 MLSTs (Figs 2 and 3). Lineage 2 contains 11 serotypes, of which 55% (6 out of 11) are not covered by PCV13, and 17 MLSTs. Lineage 3 contains 2 serotypes and 9 MLSTs.

Two NVT clades have expanded in recent decades: a clade dominated by serotype 24F isolates within Lineage 2, which also includes a basal group of serotype 15A isolates, and a serotype 11A clade within Lineage 1. The 24F clade, which we hereafter refer to as the GPSC6-24F clade, consists of 296 serotype 24F isolates, 6 isolates identified as serogroup 24 and 3 serotype 24A isolates. Three hundred and two out of three hundred and five (99%) isolates in GPSC6-24F were identified as ST162. This GPSC6-24F clade is estimated to have emerged around 1982.0 (CI: 1976.7; 1987.7) (Fig. 2) and consists of isolates from Norway (*n*=129), France (*n*=160), Denmark (*n*=15) and India (*n*=1). The 11A clade (GPSC6-11A) contains samples from Cambodia (*n*=28) and Norway (*n*=2). Twenty-eight out of thirty (93%) of the isolates in GPSC6-11A, including the 2 Norwegian isolates, were identified as ST166.

#### Antimicrobial resistance

Predicted antibiotic resistance profiles differed substantially across GPSC6 lineages (Fig. 3, Table S1). Lineage 1 contained the highest *β*-lactam non-susceptibility under meningitis breakpoints: PEN resistance (93%) and CXM resistance (91%), while CRO and CTX were predominantly intermediate (both 87%). Under NM criteria, most isolates were susceptible to PEN (94%) and third-generation cephalosporins (97% for CRO; 97% for CTX). AMO susceptibility was high (95%), whereas MEM was mainly intermediate (85%). Lineage 1 also carried a substantial multidrug resistance, with 92% classified as MDR (non-susceptible to ≥3 antimicrobial classes) (Table S5). Within Lineage 1, the 11A clade followed a similar *β*-lactam resistance pattern (Table S2): 100% PEN resistance (meningitis breakpoints) and 97% CXM resistance, alongside predominantly intermediate CRO/CTX under meningitis breakpoints (97% for each). Lineage 1 showed near-universal Trimethoprim (TMP) and Sulfamethoxazole (SMX) resistance (TMP 99%; SMX 98%) (Table S3), exemplified by the 11A clade (TMP 100% R; SMX 100% R) (Table S4; Fig. 3). The 11A clade was universally MDR (100%) (Table S5).

The vast majority of Lineage 2 isolates were predicted to be susceptible to *β*-lactams (Fig. 3, Table S1). PEN susceptibility was 94% at the meningitis breakpoint (6%) and 100% at the NM breakpoint. For each of the antibiotics CRO, CTX, AMO and MEM, 99–100% of Lineage 2 were susceptible at both breakpoints. Only 4% of Lineage 2 isolates were non-susceptible to CXM (2% I, 2% R). Consistent with this, MDR was uncommon in this lineage (6% MDR) (Table S5). The 24F clade within this lineage mirrored the same near-complete *β*-lactam susceptibility (generally ≥99% across agents, with only 1% of isolates resistant to PEN and CXM). Lineage 2 also carried substantial TMP/SMX resistance (TMP 78%; SMX 79%) (Table S3), driven by the 24F clade, which was uniformly resistant to both TMP and SMX (100% for each) (Table S4; Fig. 3). GPSC6-24F also contained little MDR (4%) (Table S5).

Lineage 3 isolates were predicted to be largely resistant to *β*-lactams at meningitis breakpoints (Fig. 3, Table S1): they were universally resistant to PEN (100%), predominantly resistant to CRO (88% R; 9% I; 4% S) and intermediately resistant to CTX (89% I; 4% R; 7% S). Using NM criteria, these same drugs shifted to mostly intermediate or susceptible calls (PEN 89% I; 11% S, CRO 88% I; 12% S, CTX 96% S; 4% R). CXM resistance was almost universal (98%), while the lineage remained susceptible to AMO (98%), and MEM was largely intermediate (91%). Lineage 3 exhibited lower levels of TMP and SMX resistance (TMP 21%; SMX 43%), with a much larger susceptible fraction than Lineages 1–2 (Table S3; Fig. 3). Ninety‑eight per cent of Lineage 3 was MDR (Table S5).

### Population dynamics and strain transmission

**Fig. 4.**
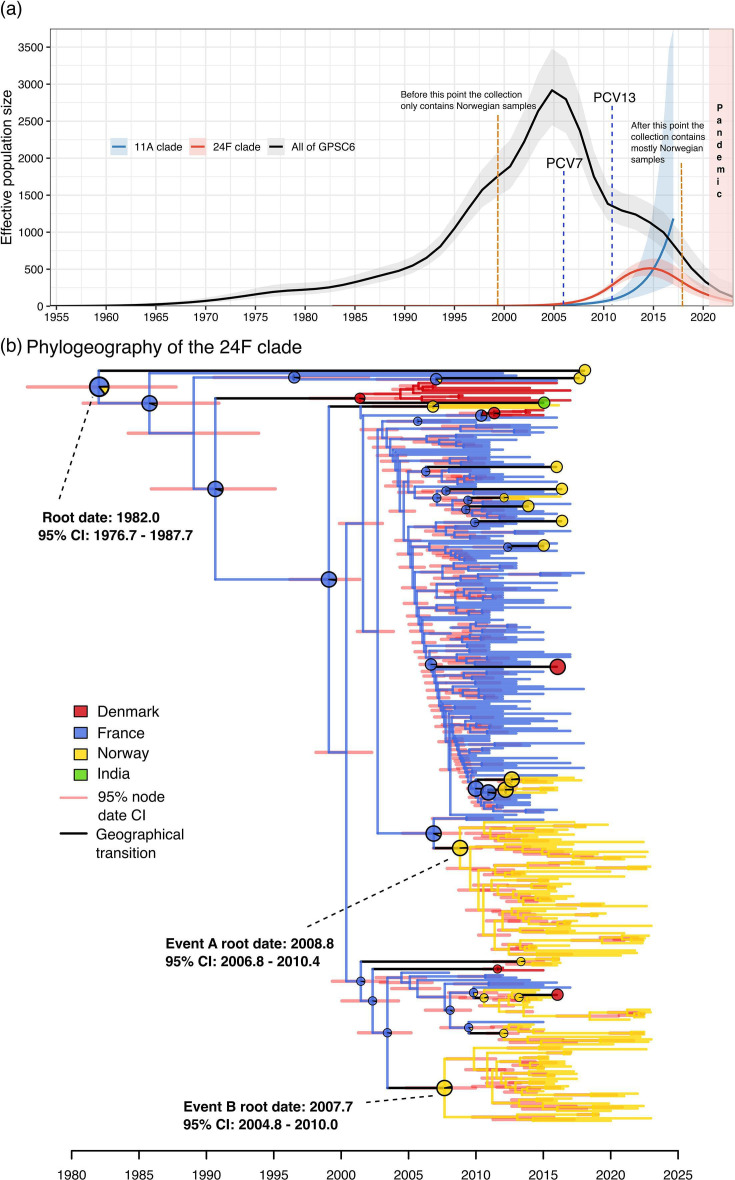
**(a**) Effective population size of GPSC6, GPSC6-24F and GPSC6-11A over time. Skygrowth estimates of effective population size (solid lines) with 95% credible intervals (shaded ribbons) are shown for GPSC6 (black/grey), the GPSC6-24F clade (in red) and the GPSC-11A clade (in blue). Vertical blue dashed lines mark the roll-out of PCVs in the Childhood Immunisation Programme – PCV7 in 2006 and PCV13 in 2011 – in Norway. Orange dashed lines indicate points where the dataset shifts to mostly Norwegian sampling (left: before this point, only Norwegian samples; right: after this point, predominantly Norwegian samples). The red-shaded period denotes the COVID-19 pandemic, during which respiratory infections in Norway (e.g. influenza) were exceptionally low, and IPD also fell markedly. (**b**) Dated phylogeographic reconstruction of the GPSC6-24F expansion. The red shaded intervals display 95% credibility intervals from BactDating, and the pie charts on the nodes represent the estimated probability of each geographical location. Black branches highlight estimated geographical state changes. Circular nodes at the tips indicate singletons, which are individual isolates at the tips of the phylogeny that are observed in a new geographical location compared to estimates on ancestral nodes. The two main import events to Norway are annotated as ‘Event A’ and ‘Event B’.

To elucidate the transmission and genetic diversity of GPSC6 over time, we estimated the effective population size for GPSC6 as a whole, as well as two NVT clades: (1) GPSC6-24F clade and (2) GPSC6-11A clade (Figs 4a and S2). Both approaches indicate an increasing trend in the effective population size of global GPSC6 over time. However, a noticeable decline was observed around the mid-2000s, coinciding with the introduction of the PCV7 vaccine in several of the countries in the dataset. PCV7 covers 4 out of the 13 serotypes observed in GPSC6, which constitute 59.3% of the isolates (Table 1). A total of 97.4% of isolates with a collection date before 2006 were covered by PCV7 and, in the period after this, fell to 48.8%.

**Table 1.** Frequency and coverage of serotypes in the GPSC6 collection by different PCVs. Shading of the cells indicates coverage by the PCV in the respective column (light grey=covered; dark grey=not covered)

Serotype	% of isolates in GPSC6 (n)	Covered by PCV7	Covered by PCV13	Covered by PCV15	Covered by PCV20
14	36.1 (351)	Yes	Yes	Yes	Yes
24F	30.5 (296)	No	No	No	No
9V	21.8 (212)	Yes	Yes	Yes	Yes
11A	3.7 (36)	No	No	No	Yes
15A	2.0 (19)	No	No	No	No
19A	1.8 (17)	No	Yes	Yes	Yes
19F	1.0 (10)	Yes	Yes	Yes	Yes
15B/15C	0.7 (7)	No	No	No	Yes
23A	0.6 (6)	No	No	No	No
Serogroup 24	0.6 (6)	No	No	No	No
23F	0.4 (4)	Yes	Yes	Yes	Yes
24A	0.3 (3)	No	No	No	No
35B	0.2 (2)	No	No	No	No
6A	0.1 (1)	No	Yes	Yes	Yes
15C	0.1 (1)	No	No	No	Yes
23B	0.1 (1)	No	No	No	No

The effective population size of GPSC6-24F has been on a continuous upward trajectory since its emergence in the 1980s until around 2015 (Figs 4a and S2). After 2017, the collection contains far fewer samples from countries other than Norway, and the effective population size of the GPSC6-24F clade declines. Since the collection mainly contains Norwegian isolates after 2017, the declining effective population size of GPSC6-24F is likely a sampling artefact. Similarly, the 11A clade has been expanding after the introduction of the PCVs. Together, these analyses suggest that the effective population size of GPSC6 declined during 2005–2016 following PCV rollout, whereas the NVT clades GPSC6-24F and GPSC6-11A expanded, likely because they were not covered by the PCVs administered during this period.

### Geographical transmission dynamics in the GPSC6-24F clade

To investigate the geographical transmission dynamics in the GPSC6-24F clade and its importation dynamics into Norway, we reconstructed the geographical states using ASR on the dated clade, based on the countries of origin of the isolates (Fig. 4b). The ASR suggests that the Norwegian isolates in the GPSC6-24F clade can largely be attributed to two import events ‘A’ and ‘B’, both having arrived in Norway by 2008–2009: Event A: 2008.83 (95% CI: 2006.8; 2010.4); Event B: 2007.7 (95% CI: 2004.8; 2010.0) (Fig. 4b). This timing coincides with the introduction of PCV7 in 2006 in Norway. In the reconstruction, the source location of introductions A and B was identified as France. It should be noted that collection bias likely has a significant impact on this analysis. Apart from Norway, France is the country with the deepest sampling, and many other European and Scandinavian countries are not represented in the collection; therefore, the identified source locations should be interpreted cautiously.

We employed LineageHomology to examine the relative contributions of import and local transmission in Norway. From this analysis, we estimate that among the 129 Norwegian GPSC6-24F isolates, there are 16 import events (95% CI accounting for ASR uncertainty: 16, 19) and 113 local transmission events (CI accounting for ASR uncertainty: 112, 115) descending from these imports. The majority of the local transmissions can be attributed to events A and B described above. Thus, the Norwegian epidemic consists of distinct 24F lineages, which likely originated from different importation events followed by local establishment and transmission.

## Discussion

Our study provides new insights into the evolutionary history and post-vaccine dynamics of *S. pneumoniae* GPSC6, with a particular focus on its emerging NVTs. Using a recombination-masked, time-scaled phylogeny, we show that serotype 24F forms a monophyletic clade inside GPSC6 with a most recent common ancestor around 1982. The 24F clade almost exclusively contained European isolates, including closely related Norwegian, Danish and French genomes (and one genome from India). The effective population size (*N_e_*) of GPSC6 started declining in the mid-2000s – coincident with PCV7 roll-out. In contrast to the overall contraction of GPSC6, the effective population sizes of the 24F and 11A sub-clades, not covered by available PCVs, started to increase. This observation is consistent with post-vaccine serotype replacement dynamics and aligns with reports from other countries of rising 24F following PCV13 [10,15], albeit often driven by other strains (e.g. GPSC10) in higher-antibiotic settings.

### GPSC6 lineages occupy different epidemiological niches

We demonstrate that GPSC6 comprises distinct lineages with different epidemiological properties. Lineage 1 overlaps with PMEN3 (ST156) described in D’Aeth *et al.* [63]. In that study, ancestral acquisition of altered PBP proteins in PMEN3 was followed by expansion of the ST156 clade during the 1980s, continuing into the early 2000s, after which it plateaued. Here, we show that as PMEN3 plateaued, another GPSC6 lineage GPSC6-24F clade (ST162) began to expand, likely because it was not covered by the PCVs in use at the time. Although the GPSC6-24F clade within Lineage 2 does not share the MDR profile of Lineage 1/PMEN3 (4% vs 92%), serotype 24F has been associated with meningitis in children [9,11] and is of high concern. In addition, like Lineage 1, the GPSC6-24F clade is uniformly resistant to trimethoprim and sulphamethoxazole, consistent with previous observations of 24F-ST162 in Norway [17]. By contrast, Lineage 3 shows lower resistance to trimethoprim and sulphamethoxazole, is predominantly MDR (98%) and is largely covered by PCVs.

### European circulation and importations to Norway

Phylogeographic reconstruction indicates multiple importations of GPSC6-24F into Norway, with two events around 2008–2009 seeding most subsequent local transmission. While France was inferred as the most likely source in our ASR, we caution that this likely reflects data availability and genome upload bias rather than true origin, a known bias in ancestral reconstructions when sampling is uneven [64]. Together with earlier regional studies – e.g. the Danish rise of 24F associated with ST162 [14] – our results support sustained GPSC6-24F circulation across northern Europe and repeated cross-border transmission.

### Vaccine era dynamics

The mid-2000s decline in GPSC6 effective population size likely reflects the removal of vaccine-type serotypes (e.g. 9V, 14 and 19F) following PCV7 introduction, with subsequent expansion of NVTs such as 24F within the strain. This pattern mirrors broader European evidence of serotype replacement after PCV10/13, where strain composition shifted, and non-PCV13 serotypes gained prominence from ~2011 onward [8]. In Norway, a recent study shows that this expansion was not accompanied by an increase in AMR among GPSC strains post-2007 [15], indicating that vaccine-driven ecological change, rather than AMR, was the primary force shaping population dynamics.

### AMR and lineage contrasts

*In silico* PBP typing and AMR determinant profiling suggest that Norwegian GPSC6-24F isolates remain largely susceptible to *β*-lactams and macrolides relative to MDR 24F expansions of GPSC10 reported elsewhere (e.g. France, Spain and Lebanon) [10,65]. This contrast reinforces that different GPSCs can mediate the same serotype’s rise under distinct national antibiotic treatment regimes, as highlighted by GPS Consortium and subsequent regional genomic studies [20]. Surveillance programmes should therefore track both serotypes and lineages within GPSCs to anticipate clinical risk and resistance trends.

### Implications for vaccines

After repeated introductions, GPSC6-24F has gained a foothold in Norway. While serotype 24F is absent from the paediatric PCV13/15 and PCV20 formulations used in many countries, it is included in the adult-specific 21-valent conjugate vaccine (PCV21, CAPVAXIVE), approved by the US FDA in June 2024 [66] and recommended by ACIP for adults as an option alongside PCV15 or PCV20 [67] and also approved by the European Commission in March 2025 [68]. PCV21 explicitly adds eight serotypes not covered by other licensed PCVs, including 24F. These developments are relevant for adult disease burden but will not directly affect the incidence of serotypes in children, as paediatric vaccine formulations still leave 24F unaddressed. The persistence of serotype 24F infection in children necessitates continued genomic surveillance and evaluation of higher-valency paediatric PCVs.

### Public health implications

Our findings suggest that NVTs (24F and 11A) belonging to GPSC6 have benefited from their exclusion from PCVs used in child vaccination programmes. Norway currently uses PCV13 (Prevenar 13) in their Childhood Immunisation Programme. PCV15 (Vaxneuvance) and PCV20 (Prevnar 20) are licensed for paediatric use but have not been adopted in the Childhood Immunisation Programme; and even if Norway were to transition to one of them, the additional serotype coverage would not be expected to affect GPSC6 in Norway because of the dominance of NVTs such as 24F. In Norway, most cases of 24F IPD can be attributed to a few successful importations from other European countries, followed by local transmission. The post-PCV expansion of GPSC6 NVTs underscores the need for genomic surveillance of the serotype and lineage dynamics within GPSC strains.

## Supplementary material

10.1099/mgen.0.001697 Uncited Supplementary Material 1.

## Abbreviations

AIC: Akaike information criterion
AMO: amoxicillin
AMR: antimicrobial resistance
ARC: additive RC
ASR: ancestral state reconstruction
BNPR: Bayesian Non-parametric Phylodynamics Reconstruction
CRO: ceftriaxone
CTX: cefotaxime
CXM: cefuroxime
DIC: deviance information criterion
ENA: European Nucleotide Archive
EU/EEA: European Union/European Economic Area
GPS: Global Pneumococcal Sequencing
GPSC6: Global Pneumococcal Sequence Cluster 6
GPSCs: Global Pneumococcal Sequencing Clusters
MDR: multidrug-resistant
MEM: meropenem
MLSTs: multilocus sequence types
NVTs: non-vaccine types
PBP: penicillin-binding protein
PCV: pneumococcal conjugate vaccine
PEN: penicillin
RC: relaxed clock
SMX: Sulfamethoxazole
TMP: Trimethoprim
TMRCA: Time to Most Recent Common Ancestor
