# Supplementary material for: Genomic epidemiology of Streptococcus pneumoniae GPSC6: post-vaccine expansion of β-lactam-susceptible serotype 24F in Europe
Source: Microb Genom. 2026 May 15;12(5):001697. doi: 10.1099/mgen.0.001697 (PMC13178296; doi:10.1099/mgen.0.001697)
Supplement: Uncited Supplementary Material 1. [file mgen-12-01697-s001.pdf]

Appendix 1: Supplementary Methods and Figures for «*Genomic Epidemiology of Streptococcus pneumoniae GPSC6: Post-Vaccine Expansion of β-Lactam-Susceptible Serotype 24F in Europe*»

Table of contents

**SUPPLEMENTARY METHODS**	**Page**
Calculation of multidrug resistance and mapping to antimicrobial classes	1
**SUPPLEMENTARY TABLES**	
Table S1. AMR profiles by drug and lineage	2 - 3
Table S2. AMR profiles by drug and clade	3 - 4
Table S3. Non-susceptibility predicted from non-β-lactam AMR determinants by drug and lineage	5 - 6
Table S4. Non-susceptibility predicted from non-β-lactam AMR determinants by drug and clade	6 - 8
Table S5. Distribution of non-susceptibility to antibiotic classes by lineage and clade	8
**SUPPLEMENTARY FIGURES**	
Figure S1. Root-to-tip analysis of the global collection	9
Figure S2. Effective population size estimates from BNPR	10

SUPPLEMENTARY METHODS

Calculation of multidrug resistance

We defined multidrug resistance (MDR) as non-susceptibility to ≥3 distinct antimicrobial classes. For each isolate and each class, the class was considered non-susceptible if any drug mapped to that class is non-susceptible (R or I was treated as non-susceptible for MDR). For determinants-based phenotype from Pathogenwatch AMR (e.g., Erythromycin, Tetracycline, Fluoroquinolones), NOT_FOUND was treated as susceptible (no resistance determinant detected). Trimethoprim, Sulfamethoxazole, and Co-trimoxazole were mapped to a single class (cotrimoxazole), so they contribute at most one class toward MDR. The class mapping is shown in the table below.

Class mapping summary

AMR Group	Antimicrobial class	Data columns
Non-β-lactams	aminoglycosides	Kanamycin
Non-β-lactams	chloramphenicol	Chloramphenicol
Non-β-lactams	cotrimoxazole	Co-trimoxazole, Trimethoprim, Sulfamethoxazole
Non-β-lactams	fluoroquinolones	Fluoroquinolones
Non-β-lactams	lincosamides	Clindamycin
Non-β-lactams	macrolides	Erythromycin
Non-β-lactams	oxazolidinones	Linezolid
Non-β-lactams	tetracyclines	Tetracycline
β-lactams	carbapenems	Meropenem (single threshold)
β-lactams	cephalosporins	Ceftriaxone (meningitis), Ceftriaxone (non-meningitis), Cefotaxime (meningitis), Cefotaxime (non-meningitis), Cefuroxime (single threshold)
β-lactams	penicillins	Penicillin (meningitis), Penicillin (non-meningitis), Amoxicillin (single threshold)

SUPPLEMENTARY TABLES

Table S1. AMR profiles by drug and lineage

Susceptible (S), intermediate (I), and Resistant (R), percentages are among isolates with a result for that antibiotic. Results are split by lineage: Lineage 1 (n=509), Lineage 2 (n=407), Lineage 3 (n=56).

Lineage	Drug	S/I/R
Lineage 1	Penicillin (meningitis)	S 7%	I 0%	R 93%
Lineage 1	Penicillin (non-meningitis)	S 94%	I 6%	R 0%
Lineage 1	Ceftriaxone (meningitis)	S 10%	I 87%	R 3%
Lineage 1	Ceftriaxone (non-meningitis)	S 97%	I 2%	R 1%
Lineage 1	Cefotaxime (meningitis)	S 10%	I 87%	R 3%
Lineage 1	Cefotaxime (non-meningitis)	S 97%	I 1%	R 2%
Lineage 1	Amoxicillin (single threshold)	S 95%	I 1%	R 4%
Lineage 1	Cefuroxime (single threshold)	S 8%	I 0%	R 91%
Lineage 1	Meropenem (single threshold)	S 10%	I 85%	R 4%
Lineage 2	Penicillin (meningitis)	S 94%	I 0%	R 6%
Lineage 2	Penicillin (non-meningitis)	S 100%	I 0%	R 0%
Lineage 2	Ceftriaxone (meningitis)	S 99%	I 1%	R 0%
Lineage 2	Ceftriaxone (non-meningitis)	S 100%	I 0%	R 0%
Lineage 2	Cefotaxime (meningitis)	S 99%	I 1%	R 0%
Lineage 2	Cefotaxime (non-meningitis)	S 100%	I 0%	R 0%
Lineage 2	Amoxicillin (single threshold)	S 100%	I 0%	R 0%
Lineage 2	Cefuroxime (single threshold)	S 97%	I 2%	R 2%
Lineage 2	Meropenem (single threshold)	S 99%	I 1%	R 0%
Lineage 3	Penicillin (meningitis)	S 0%	I 0%	R 100%

Susceptible (S), intermediate (I), and Resistant (R), percentages are among isolates with a result for that antibiotic. Results are split by lineage: Lineage 1 (n=509), Lineage 2 (n=407), Lineage 3 (n=56).

Lineage	Drug	S/I/R
Lineage 3	Penicillin (non-meningitis)	S 11% | I 89% | R 0%
Lineage 3	Ceftriaxone (meningitis)	S 4% | I 9% | R 88%
Lineage 3	Ceftriaxone (non-meningitis)	S 12% | I 88% | R 0%
Lineage 3	Cefotaxime (meningitis)	S 7% | I 89% | R 4%
Lineage 3	Cefotaxime (non-meningitis)	S 96% | I 0% | R 4%
Lineage 3	Amoxicillin (single threshold)	S 98% | I 0% | R 2%
Lineage 3	Cefuroxime (single threshold)	S 0% | I 2% | R 98%
Lineage 3	Meropenem (single threshold)	S 7% | I 91% | R 2%

Table S2. AMR profiles by drug and clade

Susceptible (S), intermediate (I), and Resistant (R) percentages are among isolates with a result for that antibiotic. Results are split by clades: 24F clade (n=305), 11A clade (n=30), and Other 3 (n=634).

Clade	Drug	S/I/R
24F clade	Penicillin (meningitis)	S 99% | I 0% | R 1%
24F clade	Penicillin (non-meningitis)	S 100% | I 0% | R 0%
24F clade	Ceftriaxone (meningitis)	S 99% | I 0% | R 0%
24F clade	Ceftriaxone (non-meningitis)	S 100% | I 0% | R 0%
24F clade	Cefotaxime (meningitis)	S 99% | I 1% | R 0%
24F clade	Cefotaxime (non-meningitis)	S 100% | I 0% | R 0%
24F clade	Amoxicillin (single threshold)	S 100% | I 0% | R 0%
24F clade	Cefuroxime (single threshold)	S 99% | I 0% | R 1%

Susceptible (S), intermediate (I), and Resistant (R) percentages are among isolates with a result for that antibiotic. Results are split by clades: 24F clade (n=305), 11A clade (n=30), and Other 3 (n=634).		
Clade	Drug	S/I/R
24F clade	Meropenem (single threshold)	S 99% | I 0% | R 0%
11A clade	Penicillin (meningitis)	S 0% | I 0% | R 100%
11A clade	Penicillin (non-meningitis)	S 97% | I 3% | R 0%
11A clade	Ceftriaxone (meningitis)	S 3% | I 97% | R 0%
11A clade	Ceftriaxone (non-meningitis)	S 100% | I 0% | R 0%
11A clade	Cefotaxime (meningitis)	S 3% | I 97% | R 0%
11A clade	Cefotaxime (non-meningitis)	S 100% | I 0% | R 0%
11A clade	Amoxicillin (single threshold)	S 97% | I 0% | R 3%
11A clade	Cefuroxime (single threshold)	S 3% | I 0% | R 97%
11A clade	Meropenem (single threshold)	S 3% | I 93% | R 3%
Other	Penicillin (meningitis)	S 18% | I 0% | R 82%
Other	Penicillin (non-meningitis)	S 88% | I 12% | R 0%
Other	Ceftriaxone (meningitis)	S 24% | I 67% | R 10%
Other	Ceftriaxone (non-meningitis)	S 90% | I 9% | R 1%
Other	Cefotaxime (meningitis)	S 24% | I 73% | R 2%
Other	Cefotaxime (non-meningitis)	S 98% | I 1% | R 2%
Other	Amoxicillin (single threshold)	S 96% | I 1% | R 3%
Other	Cefuroxime (single threshold)	S 21% | I 2% | R 78%
Other	Meropenem (single threshold)	S 24% | I 72% | R 3%

Table S3. Resistance predicted from Non-β-lactam AMR determinants by drug and lineage

Susceptible (S), intermediate (I), and Resistant (R) percentages are among isolates with a result for that antibiotic. Results are split by lineage: Lineage 1 (n=509), Lineage 2 (n=407), Lineage 3 (n=56).

Lineage	Drug	S/I/R
Lineage 1	Chloramphenicol	S 100% | I 0% | R 0%
Lineage 1	Clindamycin	S 85% | I 0% | R 15%
Lineage 1	Erythromycin	S 75% | I 0% | R 25%
Lineage 1	Tetracycline	S 83% | I 0% | R 17%
Lineage 1	Co-trimoxazole	S 1% | I 1% | R 98%
Lineage 1	Trimethoprim	S 1% | I 0% | R 99%
Lineage 1	Sulfamethoxazole	S 2% | I 0% | R 98%
Lineage 1	Fluoroquinolones	S 98% | I 0% | R 2%
Lineage 1	Kanamycin	S 97% | I 0% | R 3%
Lineage 1	Linezolid	S 100% | I 0% | R 0%
Lineage 2	Chloramphenicol	S 100% | I 0% | R 0%
Lineage 2	Clindamycin	S 97% | I 0% | R 3%
Lineage 2	Erythromycin	S 94% | I 0% | R 6%
Lineage 2	Tetracycline	S 97% | I 0% | R 3%
Lineage 2	Co-trimoxazole	S 21% | I 1% | R 78%
Lineage 2	Trimethoprim	S 22% | I 0% | R 78%
Lineage 2	Sulfamethoxazole	S 21% | I 0% | R 79%
Lineage 2	Fluoroquinolones	S 100% | I 0% | R 0%
Lineage 2	Kanamycin	S 99% | I 0% | R 1%

Susceptible (S), intermediate (I), and Resistant (R) percentages are among isolates with a result for that antibiotic. Results are split by lineage: Lineage 1 (n=509), Lineage 2 (n=407), Lineage 3 (n=56).		
Lineage	Drug	S/I/R
Lineage 2	Linezolid	S 100% | I 0% | R 0%
Lineage 3	Chloramphenicol	S 100% | I 0% | R 0%
Lineage 3	Clindamycin	S 25% | I 0% | R 75%
Lineage 3	Erythromycin	S 18% | I 0% | R 82%
Lineage 3	Tetracycline	S 36% | I 0% | R 64%
Lineage 3	Co-trimoxazole	S 57% | I 21% | R 21%
Lineage 3	Trimethoprim	S 79% | I 0% | R 21%
Lineage 3	Sulfamethoxazole	S 57% | I 0% | R 43%
Lineage 3	Fluoroquinolones	S 100% | I 0% | R 0%
Lineage 3	Kanamycin	S 29% | I 0% | R 71%
Lineage 3	Linezolid	S 100% | I 0% | R 0%

Table S4. Non-β-lactam AMR determinants by drug and clade

Susceptible (S), intermediate (I), and Resistant (R) percentages are among isolates with a result for that antibiotic. Results are split by clades: 24F clade (n=305), 11A clade (n=30), and Other 3 (n=634).

Clade	Drug	S/I/R
24F clade	Chloramphenicol	S 100% | I 0% | R 0%
24F clade	Clindamycin	S 97% | I 0% | R 3%
24F clade	Erythromycin	S 96% | I 0% | R 4%
24F clade	Tetracycline	S 97% | I 0% | R 3%
24F clade	Co-trimoxazole	S 0% | I 0% | R 100%
24F clade	Trimethoprim	S 0% | I 0% | R 100%

Susceptible (S), intermediate (I), and Resistant (R) percentages are among isolates with a result for that antibiotic. Results are split by clades: 24F clade (n=305), 11A clade (n=30), and Other 3 (n=634).

Clade	Drug	S/I/R
24F clade	Sulfamethoxazole	S 0% | I 0% | R 100%
24F clade	Fluoroquinolones	S 100% | I 0% | R 0%
24F clade	Kanamycin	S 100% | I 0% | R 0%
24F clade	Linezolid	S 100% | I 0% | R 0%
11A clade	Chloramphenicol	S 100% | I 0% | R 0%
11A clade	Clindamycin	S 0% | I 0% | R 100%
11A clade	Erythromycin	S 0% | I 0% | R 100%
11A clade	Tetracycline	S 0% | I 0% | R 100%
11A clade	Co-trimoxazole	S 0% | I 0% | R 100%
11A clade	Trimethoprim	S 0% | I 0% | R 100%
11A clade	Sulfamethoxazole	S 0% | I 0% | R 100%
11A clade	Fluoroquinolones	S 90% | I 0% | R 10%
11A clade	Kanamycin	S 100% | I 0% | R 0%
11A clade	Linezolid	S 100% | I 0% | R 0%
Other	Chloramphenicol	S 100% | I 0% | R 0%
Other	Clindamycin	S 86% | I 0% | R 14%
Other	Erythromycin	S 76% | I 0% | R 24%
Other	Tetracycline	S 85% | I 0% | R 15%
Other	Co-trimoxazole	S 19% | I 3% | R 77%
Other	Trimethoprim	S 22% | I 0% | R 78%

Susceptible (S), intermediate (I), and Resistant (R) percentages are among isolates with a result for that antibiotic. Results are split by clades: 24F clade (n=305), 11A clade (n=30), and Other 3 (n=634).		
Clade	Drug	S/I/R
Other	Sulfamethoxazole	S 20% | I 0% | R 80%
Other	Fluoroquinolones	S 99% | I 0% | R 1%
Other	Kanamycin	S 91% | I 0% | R 9%
Other	Linezolid	S 100% | I 0% | R 0%

Table S5. AMR distribution by number of antibiotic classes for each lineage and clade

Percent of isolates by how many antimicrobial classes they are non-susceptible to; MDR is defined as non-susceptible to ≥3 classes. AMR to β-lactams was estimated using the Pathogenwatch SPN-PBP-AMR pipeline; AMR to non-β-lactams was estimated using Pathogenwatch AMR.

Lineage / Clade	Number of isolates	0 classes non-susceptible	1 class non-susceptible	2 classes non-susceptible	≥3 classes non-susceptible (MDR)
Lineage 1	509	0%	7%	1%	92%
Lineage 2	407	16%	75%	3%	6%
Lineage 3	56	0%	0%	2%	98%
24F clade	305	0%	96%	0%	4%
11A clade	30	0%	0%	0%	100%
Other	637	10%	8%	3%	79%

SUPPLEMENTARY FIGURES

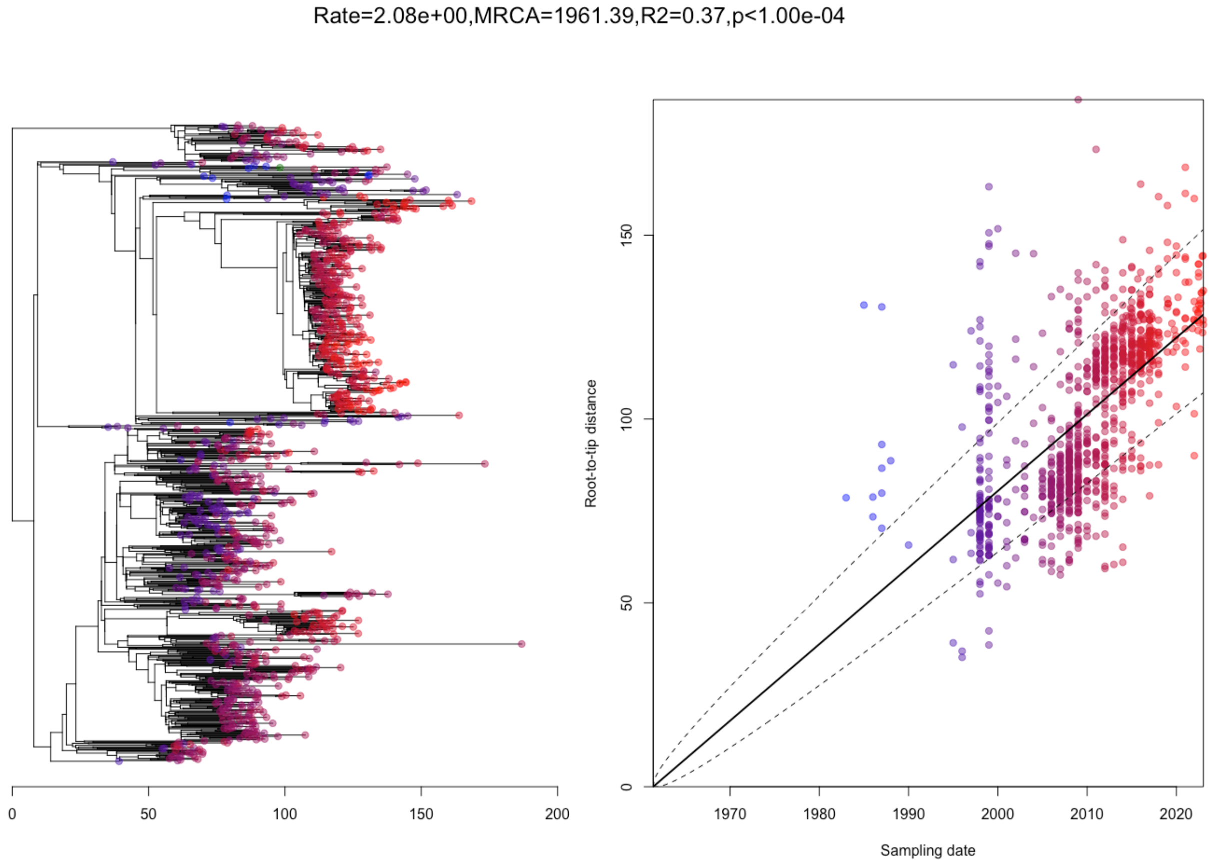

Figure S1. Root-to-tip analysis of the GPSC6 phylogeny. The left panel shows the phylogenetic tree rooted at the best root-to-tip correlation, where tip points are coloured by the time the samples were obtained (red = more recent; blue = older).

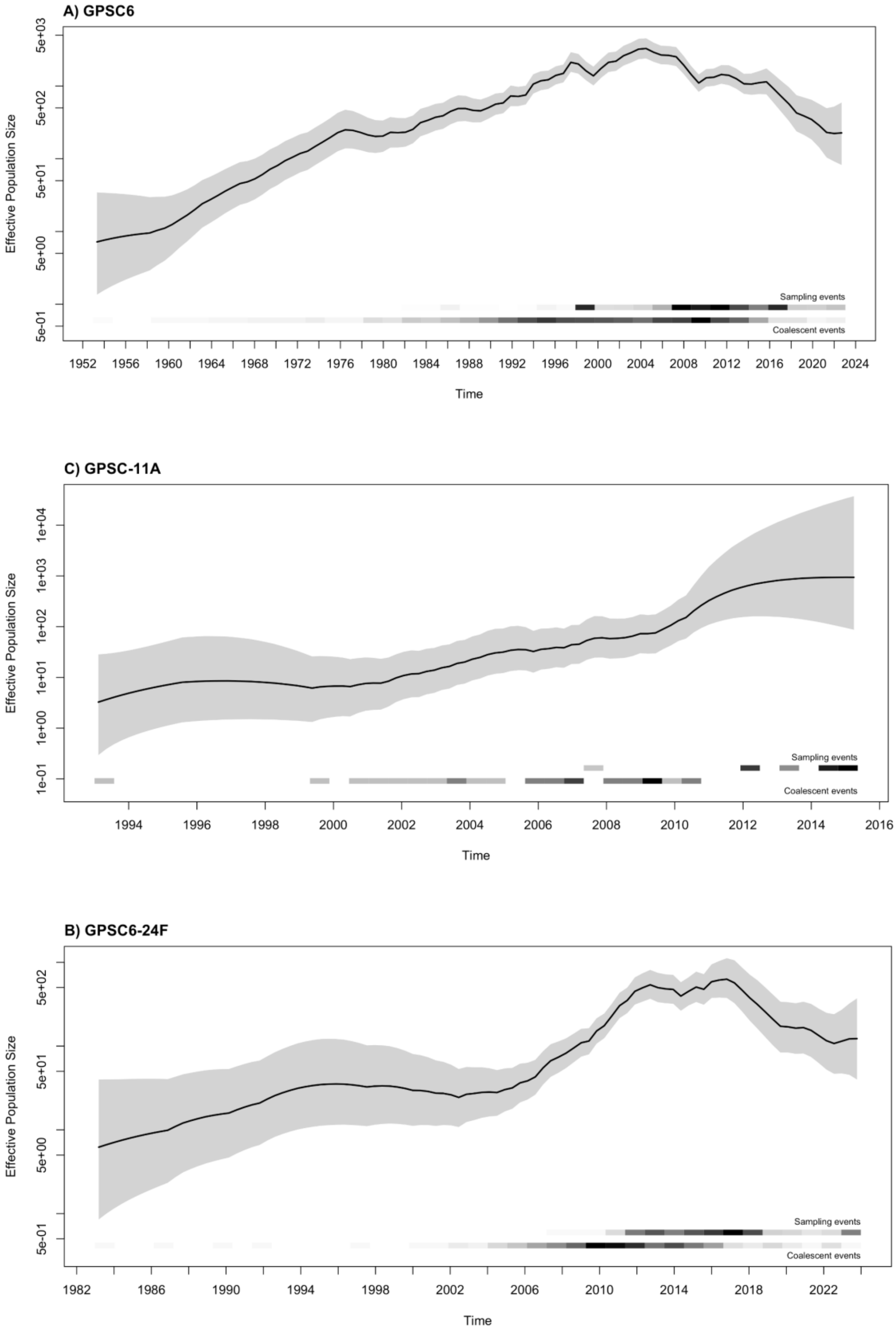

Figure S2. Effective population size estimates from BNPR for A) GPSC6, B) GPSC6-24F, and C) GPSC6-11A.
